# South Asian Ethnicity Is Related to the Highest Risk of Vitamin B12 Deficiency in Pregnant Canadian Women

**DOI:** 10.3390/nu9040317

**Published:** 2017-03-23

**Authors:** Marta Jeruszka-Bielak, Carly Isman, Theresa H. Schroder, Wangyang Li, Tim J. Green, Yvonne Lamers

**Affiliations:** 1 Food Nutrition and Health Program, Faculty of Land and Food Systems, The University of British Columbia, Vancouver, BC V6T 1Z4, Canada; marta_jeruszka_bielak@sggw.pl (M.J.-B.); carly.isman@gmail.com (C.I.); schrod10@mail.ubc.ca (T.H.S.); tina@medweight.ca (W.L.); Tim.Green@sahmri.com (T.J.G.); 2 BC Children’s Hospital Research Institute, Vancouver, BC V5Z 4H4, Canada; 3 South Australian Health Medical Research Institute, Adelaide 5000, Australia

**Keywords:** pregnancy, cobalamin, vitamin B12, low vitamin B12 status, deficiency, methylmalonic acid, MMA, ethnicity, South Asian, predictor

## Abstract

Vitamin B12 (B12) adequacy during pregnancy is crucial for maternal health and optimal fetal development; however, suboptimal B12 status has been reported in pregnant Canadian women. Methylmalonic acid (MMA) is a sensitive indicator of B12 status. Since few studies have measured MMA during pregnancy in Canadian women, the objective of this study was to evaluate B12 status in pregnant women living in Metro Vancouver, using both plasma total B12 and MMA. We recruited a convenience sample of 320 pregnant women between 20 and 35 gestational weeks from local healthcare facilities. Plasma total B12 concentrations indicative of deficiency (<148 pmol/L) and suboptimal B12 status (148–220 pmol/L) were found in 18% and 33% of the women, respectively. Normal plasma MMA concentration (<210 nmol/L) was observed in 82% of all women. Gestational age was a strong predictor of plasma total B12 and MMA concentration, and South Asian ethnicity of B-12 deficiency and MMA concentrations. Overall, there was a high discrepancy between the prevalence of B12 inadequacy depending on the biomarker used. Independently, however, South Asian women were at particular risk for B12 deficiency, likely due to lower animal source food intake. Further study of this vulnerable group and performance testing of B12 biomarkers is warranted.

## 1. Introduction

Maternal vitamin B12 (B12) adequacy is important for fetal growth and neurodevelopment, and for maternal and infant health. Low maternal B12 status has been associated with preterm birth [1], intra-uterine growth restriction [2], congenital heart defects [3], neural tube defects (NTDs) [4], and impaired cardiometabolic health in the offspring [5,6]. Maternal B12 status is a key determinant of infant B12 status [7,8]. Poor infant B12 status can have irreversible long-term consequences, including poor intellectual and cognitive performance later in childhood [9]. 

In the Canadian Health Measures Survey (Cycle 1; 2007–2009), suboptimal B12 status (serum total B12 =148–220 pmol/L) and B12 deficiency (serum total B12 <148 pmol/L) were found in 20% and 6% of adult women of all ages, respectively [10]. We previously reported a 20% prevalence of suboptimal B12 status and 14% B12 deficiency in a convenience sample of 206 reproductive-aged women of South Asian and European ethnicity living in Metro Vancouver, with a non-significant trend for a higher prevalence of B12 deficiency in South Asian women [11]. Results of two recent prospective cohort studies of pregnant Canadian women revealed a high prevalence of low B12 status in pregnancy, with deficiency as high as 38% at delivery [12,13]. 

B12 deficiency is also known to be particularly high in South Asian women, with 40%–90% of women of childbearing age in India having low serum total B12 concentration and/or elevated MMA concentration [6,14,15]. This high prevalence is attributed to dietary patterns associated with little intake—low frequency and small portion sizes—of animal source foods [6]. South Asian women of childbearing age who immigrate to high-income countries are at higher risk of B12 deficiency compared to those of European descent [11,16]. The prevalence of B12 deficiency in South Asian pregnant women living in Canada has not been reported. 

Biochemical B12 status can be assessed using direct indicators such as total B12 and holotranscobalamin, and functional biomarkers such as methylmalonic acid (MMA) and total homocysteine. Due to problems with the sensitivity and specificity of single measurements, the combined use of one direct and one functional biomarker has been recommended [17]; however, the majority of studies investigating B12 status have only measured total B12, including a previous study of pregnant women in Vancouver [13]. 

Our objective was to determine B12 status and the prevalence of B12 deficiency, using combined plasma total B12 and MMA concentrations, in a sample of healthy pregnant women in Metro Vancouver. We also aimed to identify dietary, demographic, and lifestyle factors associated with B12 status.

## 2. Materials and Methods

### 2.1. Study Population

This cross-sectional study was conducted in Metro Vancouver, British Columbia, Canada, between February 2009 and February 2010 [18]. Using convenience sampling, 340 pregnant women were recruited from BC Women’s Hospital and Health Centre, Douglas College prenatal programs, and various Vancouver Coastal Health Community Health Centres. Recruitment was conducted actively by health professionals such as nurses and dietitians at these locations, as well as passively by brochures left at clinics and advertisements in local newspapers. Women were eligible to participate if they were between the 20th and 35th week of gestation, and having a singleton pregnancy. Women were excluded from participating if they had any co-morbid conditions such as gestational diabetes, cardiac or renal disease, HIV/AIDS, chronic hypertension, or autoimmune disease. The study was approved by the University of British Columbia Children’s and Women’s Research Ethics Board (#H08-01447). Participants provided written informed consent prior to the study.

### 2.2. Demographic, Lifestyle, Dietary, and Anthropometric Variables

Data were collected at a healthcare centre during a one-time interview lasting approximately 30–60 min. Participants completed a questionnaire on demographics and lifestyle factors to collect the following information: age, week of gestation, supplement use, smoking status, self-identified ethnicity, income, and highest educational level attained. We categorized women’s ethnicity into European, Chinese Asian, South Asian, and “Other” ethnicities including participants of Korean, Japanese, Southeast Asian, African, Latin American, West Asian, and Arabian descent. Data for highest education level were trichotomized into the following categories: less than high school—no schooling or some elementary or completed elementary or some high school; high school degree—completed high school or some trade/vocational training or some university; university or trade school—completed trade/vocational training or completed university. Information on usual consumption of meat, fish, egg, and dairy products was collected using a qualitative questionnaire (yes/no). Participants were asked to provide their pre-pregnancy weight, and height was measured by a research assistant in order to calculate BMI. 

### 2.3. Biochemical Analysis

Participants were asked to provide a non-fasting blood sample, from which plasma was extracted and subsequently stored at −80 °C until further analysis. Plasma samples were analyzed for B12 biomarkers unless they were haemolysed (defined by visual assessment). Plasma total B12 concentrations were measured using Microparticle Enzyme Immunoassay technology (Abbott Laboratories, Abbott Park, IL, USA). Inter-assay variation (CV %) for the medium quality control was 3.7%. Plasma MMA concentrations were quantified using liquid chromatography–tandem mass spectrometry (LC–MS/MS) (LC: Agilent Technologies, Santa Clara, CA, USA; MS/MS: SCIEX, Framingham, MA, USA) [19]. The intra- and inter-assay variations (CV %) of the method were 0.8% and 3.0%, respectively. The use of non-fasting blood samples for determining B12 status does not pose challenges in results interpretation because the indicators plasma total B12 and MMA do not underlie postprandial changes [20,21].

### 2.4. Statistical Analysis

To date, there are no established pregnancy-specific cutoffs for B12 biomarkers. Vitamin B12 status was categorized using the non-pregnant adult cutoffs for plasma total B12: B12 deficiency: <148 pmol/L; suboptimal B12 status: 148–220 pmol/L; B12 adequacy: >220 pmol/L. Plasma MMA concentrations were categorized as follows: <210 nmol/L, B12 replete; 210–370 nmol/L, mildly elevated MMA concentration; >370 nmol/L functional B12 deficiency [22,23]. 

Because plasma total B12 and MMA concentrations were skewed, non-parametric tests were applied and results are presented as medians and interquartile ranges (IQR). The impact of categorical health and lifestyle variables on total B12 concentrations was assessed using the Mann-Whitney *U* test or the Kruskal-Wallis test. The distribution of B12 deficiency, suboptimal status, and adequacy in relation to qualitative characteristics was examined with chi-squared tests using Bonferroni adjustment. Spearman’s Rank correlation was used to examine the association between the biomarkers. Multiple linear and logistic regression analyses were applied to identify predictors of plasma total B12 and MMA concentrations, and predictors of B12 deficiency (dichotomized; defined as plasma total B12 <148 pmol/L) and mildly elevated MMA concentration (dichotomized; defined as plasma MMA >210 nmol/L [22]), respectively. Residuals were examined for heteroskedasticity and normality. No obvious heteroskedasticity was observed, and there were only minor deviations of the residuals from normality, which was mitigated by the large sample. Therefore, no transformation of the plasma total B12 and MMA concentrations was performed. Backwards model selection based on Akaike’s Information Criterion (AIC) was used to construct the best-fit model to predict plasma total B12 or MMA concentration, B12 deficiency, or elevated MMA concentration. Prior to entry into the models, collinearity of the potential predictor variables was assessed. Statistical significance was defined as *p* < 0.05. Statistical analyses were conducted using Stata 12.1 (StataCorp LLC, College Station, TX, USA) for Windows 10 (Microsoft Corp., Redmond, WA, USA).

## 3. Results

### 3.1. Participant Characteristics 

Biomarker results were obtained from 320 pregnant women; the characteristics of these participants have been previously reported [18]. Briefly, the median age was 31 years (range 16–47 years) and 63% of the participants were >30 years. Median gestational age was 30 weeks and over two-thirds of the participants were in their third trimester of pregnancy (≥27 gestational weeks). Women had a median pre-pregnancy BMI of 22.5 kg/m^2^; 73% were classified as normal weight, 20% as overweight and 7% as obese. Almost half of the participants were of European descent (47%), 19% Chinese, 8% South Asians, and the remaining 26% of “other” ethnicities. The majority of women (57%) had a university degree and 6% reported to have used tobacco during pregnancy. 

### 3.2. Plasma Total B12 and MMA Concentration

The median (IQR) plasma total B12 concentration was 215 (160, 283) pmol/L (Table 1). Plasma concentrations indicative of B12 deficiency were found in 18% of women and 33% had suboptimal B12 status (Table 1). The highest prevalence of B12 deficiency, 61.5%, was observed in women of South Asian ethnicity. Over 90% of the participants reported taking B12-containing supplements. Usual consumption of fish, meat, eggs, and dairy products was reported by 74%, 83%, 95% and 99% of the women, respectively. Plasma total B12 concentrations were higher in B12 supplement users and women with higher education. Women of South Asian ethnicity had lower plasma total B12 concentration (Table 1).

The median (IQR) plasma MMA concentration was 140 (110, 187) nmol/L. The majority of pregnant women (82%) had plasma MMA concentrations indicative of B12 adequacy (<210 nmol/L; [22]), and six women (1.9%) had functional B12 deficiency (defined as having elevated MMA concentrations of >370 nmol/L; [23]). Overt B12 deficiency (defined as plasma total B12 <148 pmol/L and concurrent plasma MMA >370 nmol/L) was present in five out of the 320 women (1.6% of the study population), with three women of South Asian ethnicity. The remaining women with plasma B12 concentrations indicative of B12 deficiency had either normal (*n* = 31) or marginally elevated (*n* = 22) MMA concentrations.

There was a significant negative correlation between plasma total B12 and MMA concentrations (*ρ* = −0.38, *p* < 0.0001). Women with plasma MMA concentrations <210 nmol/L had significantly higher plasma total B12 concentrations (median 229; IQR 176, 297 pmol/L) compared to women with mildly elevated (median 157; IQR 114, 212 pmol/L) and elevated MMA concentration (median 132; IQR 107, 209 pmol/L) (*p* < 0.0001). 

Plasma MMA concentrations were significantly higher in pregnant women of South Asian descent (median 223; IQR 131, 288 nmol/L) compared to European (median 144; IQR 115, 180 nmol/L), Chinese Asian (median 135; IQR 103, 190 nmol/L), and other ethnic groups (median 137; IQR 104, 174 nmol/L) (*p* = 0.004). Plasma MMA concentrations were significantly higher in third-trimester (median 146; IQR 117, 199) compared to second-trimester (median 128; IQR 96, 168) pregnant women (*p* < 0.001). There was no difference in plasma MMA concentrations with respect to pre-pregnancy BMI, supplement use, fish and meat intake, smoking, education, or income. 

### 3.3. Predictors of B12 Status

There were complete data for multivariate analyses of 284 women after exclusion of 36 cases with missing data for meat intake and an additional case with missing data for education. The income variable was missing for 100 participants (~35%) and therefore not included in the modelling. Fish, egg, and meat intake were highly collinear with 205 out of 284 women reporting the consumption of all three. Similarly, there was a relationship between ethnicity and fish consumption with Europeans (106/140 = 76%), “Other” (54/66 = 82%), and Chinese Asian (43/56 = 77%) reporting more fish consumption than South Asians (9/22 = 41%). No similar relationship was observed between ethnicity and meat or egg consumption; however, very few women report no egg consumption (5%) or meat consumption (6%). We thus included ethnicity but excluded fish intake from the initial full model during model selection. 

For plasma total B12, backwards model selection resulted in five variables remaining in the best-fit linear regression model: age, gestational age, pre-pregnancy BMI, education, and use of B12 supplements (Table 2); and two variables remaining in the best-fit logistic regression model, ethnicity and use of B12 supplements (Table 3). For plasma MMA, backwards model selection resulted in four variables remaining in the best-fit linear regression model: gestational age, ethnicity, smoking during pregnancy, and egg consumption (Table 4); and three variables remaining in the best-fit logistic regression model, gestational age, ethnicity, and egg intake (Table 5).

Plasma total B12 concentration was positively associated with age, education, and B12 supplement use (Table 2). Conversely, there was a negative relationship between gestational age and pre-pregnancy BMI with plasma total B12 concentration. Multivariate logistic regression revealed that the odds of being B12 deficient were about 10-times higher for those who self-identified as South Asians relative to those who identified as European (with “Other” and Chinese Asian ethnicity not statistically different from European ethnicity) (Table 3). The odds of being B12 deficient were 69% lower for those who were taking B12 supplements.

Plasma MMA concentration was positively associated with gestational age, ethnicity, and smoking. South Asian women had higher MMA concentration on average than European, with no discernible difference between the other ethnicities. Conversely, there was a negative relationship between plasma MMA and egg intake; however, because egg intake was nearly universal, this should be interpreted with caution. The odds of having plasma MMA concentration >210 nmol/L were about 5-times higher for those who self-identified as South Asian compared to those who self-identified as European (with Chinese Asian and “Other” ethnicities not statistically different from European). For every increase in one week of gestational age, the odds of having MMA >210 nmol/L were increased by about 11%.

## 4. Discussion

Vitamin B12 sufficiency in pregnancy is essential for maternal and fetal wellbeing. Assessment of B12 status of pregnant women living on the west coast of Canada using both plasma total B12 and MMA has not previously been conducted. In light of this, we measured plasma total B12 and MMA in 320 pregnant women living in Vancouver, British Columbia. We found that 33% of the women had suboptimal B12 status and 18% were B12 deficient using total B12 as sole indicator. Overt B12 deficiency, defined as plasma total B12 <148 pmol/L and concurrent plasma MMA >370 nmol/L, was uncommon and found in only 1.6% of the study population. We observed lower total B12 and higher plasma MMA concentrations in South Asian women compared to other ethnicities, and determined that the odds of being B12 deficient and having elevated MMA concentration were about 10-times and 5-times higher, respectively, for those who self-identified as South Asians relative to those who identified as European. The odds of being B12 deficient were 69% lower for those who were taking B12 supplements. 

Comparing the results of studies investigating B12 status among different populations presents a number of challenges. Firstly, a wide variety of markers are used to measure B12 status, including serum/plasma total B12, MMA, total homocysteine, holotranscobalamin and total transcobalamin, and haptocorrin [24,25]. Secondly, different laboratory methods are used even for the same biomarker; for example, total B12 can be measured using microbiological [26] or immunoassays [10]. Thirdly, different cutoffs have been applied to define deficiency (e.g., for serum total B12 <125 pmol/L [27], <148 pmol/L [10,28], or even <180 pmol/L [29]). The cutoff for MMA, 210 nmol/L, was the reference value (95th percentile) of B12-replete adults with normal renal function (determined by serum creatinine) in the NHANES [22]. MMA concentrations >370 nmol/L is the “generally agreed on cutoff for elevated plasma MMA” [22] and was derived from the reference value (97.5th percentile) of healthy adults aged 40–68 years [30]. To date, there are no established cutoffs for B12 status during pregnancy for any of the B12 biomarkers. Accordingly, study findings should be compared with caution.

Two recent Canadian studies have reported the prevalence of B12 deficiency in pregnancy, with varying results. In the PREFORM study in Toronto (*n* = 368), the prevalence of suboptimal B12 status (serum total B12 <210 pmol/L) was 35% at 12–16 gestational weeks and 43% at delivery, and the prevalence of B12 deficiency (serum total B12 <148 pmol/L) was 17% and 38%, respectively [12]. In Vancouver, the prevalence of suboptimal B12 status and B12 deficiency was 21% and 10% in 264 women at 16 gestational weeks, and 35% and 23% in 220 women at delivery, respectively [13]; plasma MMA concentration was not measured in the study by Wu et al. [13]. In the PREFORM study, plasma MMA concentration was measured and the prevalence of functional B12 deficiency (defined as MMA >271 nmol/L) was two percent in early pregnancy and five percent at delivery [12]. In the present study, 26 (eight percent) of second- and third-trimester pregnant women had plasma MMA concentration >270 nmol/L. Notwithstanding the variable findings and the need for performance testing of B12 biomarkers during pregnancy, the prevalences of low total B12 concentration in pregnant Canadian women are high and highlight the importance of measuring B12 status early in pregnancy in order to develop interventions and prevent adverse maternal and fetal health outcomes. 

In the present study, plasma MMA concentrations were positively associated with gestational age in these women. Differences in plasma total B12 concentrations were less pronounced and negatively correlated to gestational age. Because median MMA concentrations of women in their second (128 nmol/L) and third trimester (146 nmol/L) were well within the normal range (i.e., <210 nmol/L), the relationship we found between gestational age and MMA concentration does not appear to be clinically significant. Our findings are consistent with those of Murphy et al. [31], who reported increases in plasma MMA between the first and third trimester. In a longitudinal study in 406 Danish pregnant women, the prevalence of plasma total B12 concentrations <150 pmol/L (i.e., B12 deficiency) also increased over pregnancy, from 15% at 18 weeks of gestation, to almost 43% at 39 weeks of gestation [32]. Another prospective longitudinal study of pregnancy indicated that the prevalence of B12 deficiency increased between the second and third trimester from 8% (*n* = 3) to 35% (*n* = 12) in healthy pregnant women with B12 intake >RDA (2.6 µg/day) [28] supporting the need for pregnancy-specific cutoffs. The decrease in plasma total B12 during pregnancy could result from increased metabolic rate, active B12 transport across the placenta, and hemodilution. This suggestion is supported by the finding that plasma total B12 concentrations significantly increase postpartum, without supplementation [32,33]. Altering cutoffs for B12 biomarker concentrations in pregnant women will require extensive research to ensure the prevention of adverse health outcomes. 

In our study, women who did not take supplements had double the risk of B12 deficiency compared to those who took B12-containing supplements (Table 3). Plasma total B12 concentration substantially differed between supplement users and non-users (Table 1); however, supplement use was not a significant predictor of plasma total B12 concentration (Table 2), which might be explained by the low number (*n* = 23/320; 7%) of women not using supplements. The prevalence of supplement use in pregnant Canadian women was reported to be very high with 89% of women using supplements in late pregnancy [34]. The B12 content in prenatal supplements in Canada greatly varies from 2 µg/day to 12 µg/day; the majority (62%) of supplement users in this study reported to use prenatal supplements with a dosage of 2.6 µg/day. Supplement use however was neither a predictor of plasma MMA concentrations nor associated with a lower risk for having elevated MMA. This finding might indicate that the majority of the women in this study were B12 replete, as reflected by their MMA concentration, and supplemental B12 only affected circulating total B12. This would support the hypothesis and B12 absorption findings by Greibe et al. [33] that the decrease in circulating total B12 concentration throughout gestation does not reflect B12 deficiency but ‘normal’ pregnancy-related changes in total B12 that do not impair intracellular B12 status.

An important finding of our study is that the majority of South Asian pregnant women in our cohort had suboptimal B12 status (11.5%) or were B12 deficient (61.5%). Moreover, three out of five women with overt B12 deficiency were of South Asian ethnicity. Our findings are comparable to those of a recent study on pregnant women from urban South India reporting that 51% had plasma total B12 <150 pmol/L and 76% had elevated MMA concentrations (defined as plasma MMA >260 nmol/L; compared to 46% in the current study) [15]. Of those women, 42% had impaired B12 status (i.e., total B12 <150 pmol and MMA >260 nmol/L). The study found that not consuming yogurt or fish and being primiparous were predictors of poor B12 status [15]. Cultural and religious practices that promote vegetarianism inherent to certain Indian communities around the world place these groups at greater risk of B12 deficiency during pregnancy. In a study conducted in the US, Indian women had significantly lower median serum total B12 concentrations than did “other ethnicity” (combining whites, blacks, non-Indian Asians and Latin Americans) and 24% were B12 deficient (<180 pmol/L) [29]. A randomized controlled trial of B12 supplementation in South Asian immigrant women living in New Zealand reported that 48% of the cohort (*n* = 62) had serum total B12 <222 pmol/L [16]. Supplementation with 6 μg of cyanocobalamin per day for six months increased serum total B12 by 30% [16]. 

In contrast to South Asian immigrant women, our study showed that Asian pregnant women (mostly Chinese, Southeast Asian, Japanese, Korean, and Filipino) had the highest total B12 concentrations, and the lowest prevalence of B12 inadequacy. The risk of being B12 deficient was 50% lower in Asians than in women of European descent. Similar results have been observed in an elderly population, where Asian-Americans (Chinese, Korean, Vietnamese, Japanese, or Filipino origin) had significantly higher plasma total B12 concentrations compared to European whites [35]. 

Without collecting quantitative data on food product consumption, we do not know what the overall B12 intake was. In our study, meat or fish consumption did not have a significant effect on plasma total B12 concentrations. Egg and dairy intake was reported by 95% and 99%, respectively, of all women. Vogiatzoglou et al. [26] found that higher consumption of dairy products (especially milk) and fish increased plasma total B12 concentrations, but meat and eggs did not. Intake of fish, but not other animal source foods, decreased the risk of having inadequate B12 status in South Indian pregnant women [15]. Of the South Asians in the current study 59% reported eating no fish, 27% no meat, and 27% no eggs. Ninety-five percent of South Asian women used B12-containing prenatal vitamin supplements compared to 89% and 100% of European and Chinese Asian women, respectively. We hypothesize that low dietary B12 intake likely contributed to the poor B12 status observed in the South Asian women. 

In our study, pre-pregnancy BMI was negatively correlated with plasma total B12 (*ρ* = −0.184; *p* = 0.001) but did not significantly influence the median values of either marker or the prevalence of B12 deficiency. We observed that B12-replete pregnant women had a tendency to have a lower median BMI value. Pre-pregnancy BMI was the strongest independent predictor of plasma total B12 concentration, as was gestational age. First-trimester BMI was inversely associated with third-trimester serum total B12 concentration in a retrospective case-control study of 344 UK pregnant women who were not taking B12-containing supplements [36]. The BMI at 28 weeks of gestation was an independent predictor of circulating total B12 concentrations in UK pregnant women [37]. The Canadian Health Measures Survey 2007–2009 showed that the prevalence of B12 adequacy was lower in Canadian adults with obesity compared to normal weight and overweight adults of all age groups [10]. Being obese may alter B12 absorption, excretion or metabolism, but whether the relationship is casual [38] requires further investigation. 

The strength of our research is the measurement of two biomarkers for B12 status assessment as per recommendation by an expert panel [17]. We however acknowledge the limitations of our study that include the convenience sampling and the lack of a sensitive dietary assessment tool to quantify dietary B12 intake. We also acknowledge the lack of hematological indicators, such as hemoglobin, mean corpuscular volume, or blood cell count, to investigate whether overt B12 deficiency, as observed in five women in this study, was associated with clinical outcomes. The original study did not include the measurement of hematological indicators and the retrospective analysis was not feasible for reasons of biospecimen requirements. We thus cannot conclude whether the overt B12 deficiency was of clinical concern. Women with overt B12 deficiency did not differ from the other 315 women in age (30.6 years versus 31.4 years, *p* = 0.74) or BMI (24.5 kg/m^2^ versus 22.5 kg/m^2^, *p* = 0.42); but reported lower intake of fish (40% versus 75%), meat (67% versus 94%), and egg (80% versus 95%). Nutritional status can be influenced by genetic modifiers of molecules involved in the digestion, absorption or metabolism of nutrients. Genetic variants related to B12 metabolism might explain some of the variation in our findings with respect to elevated MMA [39] and low total B12 concentration [40,41]; however, because we lack the women’s consent for genotyping in this study, we were unable to investigate this.

The assessment of B12 status is challenged by the sensitivity and specificity limitations of individual biomarkers; we therefore followed the recommendations to using at least one direct and one functional indicator of B12 status [17] while respecting available sample volume and budget. We measured total B12, the most commonly used biomarker and direct indicator, and MMA, the more specific functional indicator of B12 deficiency compared to total homocysteine. The additional measurement of total homocysteine might not have allowed further insight to the B12 status of these women; in Canada, a folate-replete nation, elevated total homocysteine concentrations (defined as >13 µmol/L [22,42]) was observed in five percent of the general population [10] but not in pregnant Canadian women [12]. While MMA is considered the more specific indicator of B12 status, we acknowledge that MMA is influenced by renal function [43] and we lack the measurement of creatinine to control for renal function in the interpretation of elevated MMA concentration. Due to budget limits, we also did not include the measurement of folate that has an interdependent metabolic role with B12. The access to both total B12 and folate data would have allowed us to investigate the interaction of folate and B12 on biochemical outcomes. High folate status with low plasma total B12 concentration has been associated with higher MMA concentration in older adults [44,45,46], however not in younger adults [46]. The interaction of high folate and low B12 status on biochemical outcomes in pregnant women warrants further investigation.

## 5. Conclusions

Protecting women of reproductive age from B12 deficiency is important to the health of mothers and their offspring. Our study showed that a sample of pregnant women in Vancouver, Canada, had a high prevalence of low circulating total B12 concentrations, with 18% being classified as B12 deficient. However, overt B12 deficiency was present in only 1.6% of the sample. The observed discrepancy in the prevalence of B12 adequacy depending on the biomarker and cutoff used emphasizes the need for pregnancy-specific cutoffs and performance testing of B12 biomarkers during pregnancy. Independent of the biomarker used, individually or combined, South Asian pregnant women were at particular risk for B12 deficiency compared to other ethnic groups, and further study of this vulnerable group is required. 

## Figures and Tables

**Table 1:** Plasma total B12 concentration (pmol/L) and prevalence of B12 deficiency, suboptimal B12 status and B12 adequacy, stratified by demographic, anthropometric, lifestyle and dietary characteristics.

	*n*	Median (Interquartile Range) *	Prevalence, *n* (%)			
			<148 pmol/L	148–220 pmol/L	>220 pmol/L	*p* Value ^$^
**All participants**	320	215 (160, 283)	58 (18)	106 (33)	156 (49)	
**Age (years)**						
<30	120	185 ^a^ (145, 245)	31 (26)	41 (34)	48 (40)	0.001 ^#^
≥30	200	230 ^b^ (182, 298)	27 (14)	53 (26)	120 (60)	
**Gestational age (weeks)**						
<27	106	229 (176, 298)	16 (15)	28 (26)	62 (59)	NS
≥27	214	210 (154, 275)	42 (20)	66 (31)	106 (49)	
**Pre-pregnancy body mass index (kg/m^2^)**						
<25	235	222 (166, 291)	39 (17)	67 (28)	129 (55)	NS
25–29.9	64	204 (151, 263)	15 (23)	19 (30)	30 (47)	
≥30	21	189 (159, 243)	4 (19)	8 (38)	9 (43)	
**Ethnicity**						
European	150	203 ^a^ (160, 277)	24 (16)	61 (41)	65 (43)	<0.001 ^#^
Chinese	60	256 ^b^ (202, 311)	4 (7)	14 (23)	42 (70)	
South Asian	26	132 ^c^ (105, 231)	16 (61.5)	3 (11.5)	7 (27)	
Other	84	220 ^a,b^ (171, 269)	14 (17)	28 (33)	42 (50)	
**Education**						
Less than high school	21	172 ^‡^ (161, 209)	3 (14)	13 (62)	5 (24)	0.004 ^#^
High school degree	77	206 (147, 264)	20 (26)	21 (27)	36 (47)	
University or trade school	221	228 ^‡^ (171, 287)	35 (16)	60 (27)	126 (57)	
**Family income per year ($)**						
<40,000	36	184 (151, 230)	7 (19)	16 (44)	13 (36)	NS
40,000 to <80,000	52	223 (171, 280)	9 (17)	14 (27)	29 (56)	
80,000 to <120,000	57	231 (160, 302)	10 (17)	17 (30)	30 (53)	
≥120,000	55	211 (176, 280)	8 (14)	18 (33)	29 (53)	
Unknown	72	225 (164, 306)	13 (18)	16 (22)	43 (60)	
Not answered	48	208 (152, 271)	11 (23)	13 (27)	24 (50)	
**Smoking of tobacco during pregnancy**						
Yes	18	200 (160, 237)	3 (17)	9 (50)	6 (33)	NS
No	302	219 (160, 284)	55 (18)	85 (28)	162 (54)	
**Use of B12-containing supplements**						
Yes	297	222 ^a^ (162, 284)	50 (17)	85 (29)	162 (54)	0.002 ^#^
No	23	173 ^b^ (140, 221)	8 (35)	9 (39)	6 (26)	
**Fish consumption**						
Yes	238	223 (160, 287)	42 (18)	73 (31)	123 (52)	NS
No	82	204 (153, 258)	16 (20)	33 (40)	33 (40)	
**Meat consumption**						
Yes	267	216 (160, 280)	49 (18)	80 (30)	138 (52)	NS
No	18	204 (145, 302)	5 (28)	4 (22)	9 (50)	

NS, non-significant (*p* > 0.05), * Estimates within a column subgroup not sharing a common superscript letter are significantly different; Mann-Whitney *U* test or Kruskal-Wallis test (all *p* < 0.001) with Dunn’s test for pairwise comparison (*p* < 0.05); ^‡^ *p* = 0.065. ^$^ Chi-squared test to determine if the number of participants across categories of B12 status is random; ^#^ significant after Bonferroni correction, i.e., *p* < 0.05/(*n* of tests) = 0.05/10 = 0.005.

**Table 2:** Predictors of plasma total B12 concentration in linear regression model.

	Univariate		Multivariate	
	β Coefficient (95% Confidence Interval)	*p* Value	Adjusted β Coefficient (95% Confidence Interval)	Adjusted *p* Value
Age (years)	3.05 (0.49, 5.6)	0.02	2.07 (−0.54, 4.67)	0.12
Gestational age (weeks)	−3.12 (−5.63, −0.61)	0.02	−2.96 (−5.41, −0.51)	0.02
Ethnicity				
European	reference	0.10		
Other	−5.74 (−38.8, 27.3)			
Chinese Asian	33.8 (−1.20, 68.8)			
South Asian	−26.7 (−77.4, 24.1)			
Pre-pregnancy body mass index (kg/m^2^)	−3.75 (−6.74, −0.76)	0.01	−3.37 (−6.31, −0.42)	0.03
Smoking	−31.7 (−90.8, 27.3)	0.29		
Education				
Less than high school	reference	0.02	reference	0.13
High school degree	46.4 (−11.3, 104)		32.3 (−24.6, 89.2)	
University or trade school	69.2 (16.3, 122)		50.8 (−2.61, 104)	
B12 supplement use	43.9 (−6.4, 94.2)	0.09	42.1 (−6.92, 91.1)	0.09
Fish intake	13.2 (−17.2, 43.5)	0.39		
Meat intake	13.1 (−41.2, 67.4)	0.63		
Egg intake	41.4 (−19.5, 102.3)	0.18		

*p* values are from likelihood ratio tests.

**Table 3:** Odds Ratios (OR) for having B12 deficiency, defined as plasma total B12 concentration <148 pmol/L, in logistic regression model.

	Univariate		Multivariate	
	OR (95% Confidence Interval)	*p* Value	Adjusted OR (95% Confidence Interval)	Adjusted *p* Value
Age (years)	0.95 (0.89, 1.00)	0.07		
Gestational age (weeks)	1.03 (0.97, 1.09)	0.28		
Ethnicity				
European	reference	<0.0001	reference	<0.0001
Other	1.25 (0.57, 2.62)		1.41 (0.64, 3.04)	
Chinese Asian	0.39 (0.11, 1.08)		0.48 (0.13, 1.35)	
South Asian	8.90 (3.43, 24.7)		10.4 (3.90, 29.4)	
Pre-pregnancy body mass index (kg/m^2^)	1.05 (0.98, 1.11)	0.17		
Smoking	1.07 (0.24, 3.51)	0.92		
Education				
Less than high school	reference	0.18		
High school degree	2.01 (0.58, 9.44)			
University or trade school	1.07 (0.33, 4.79)			
B12 supplement use	0.34 (0.14, 0.91)	0.03	0.31 (0.11, 0.86)	0.03
Fish intake	0.96 (0.50, 1.95)	0.91		
Meat intake	0.59 (0.21, 1.90)	0.35		
Egg intake	0.29 (0.10, 0.91)	0.03		

*p* values are from likelihood ratio tests.

**Table 4:** Predictors of plasma methylmalonic acid (MMA) concentration in linear regression model.

	Univariable		Multivariable	
	β Coefficient (95% Confidence Interval)	*p* Value	Adjusted β Coefficient (95% Confidence Interval)	Adjusted *p* Value
Age (years)	−1.16 (−3.05 to 0.74)	0.23		
Gestational age (weeks)	2.61 (0.78 to 4.45)	0.01	2.88 (1.11 to 4.65)	0.002
Ethnicity				
European	reference	0.0007	reference	0.008
Other	−8.69 (−32.4 to 15.1)		−13.7 (−36.5 to 9.14)	
Chinese Asian	−13.8 (−39.0 to 11.3)		−11.4 (−35.7 to 13.0)	
South Asian	67.4 (30.9 to 104)		52.6 (15.8 to 89.4)	
Pre-pregnancy body mass index (kg/m^2^)	0.57 (−1.64 to 2.79)	0.61		
Smoking	32.8 (−10.4 to 76.0)	0.14	40.7 (−1.05 to 82.4)	0.06
Education				
Less than high school	reference	0.12		
High school degree	−32.1 (−74.7 to 10.5)			
University or trade school	−40.4 (−79.4 to −1.42)			
B12 supplement	−8.74 (−45.8 to 28.3)	0.64		
Fish intake	−17.8 (−40.0 to 4.39)	0.12		
Meat intake	−24.3 (−63.9 to 15.5)	0.23		
Egg intake	−101 (−145 to −58.2)	<0.0001	−72.6 (−117 to −28.2)	0.002

*p* values are from likelihood ratio tests.

**Table 5:** Odds Ratios (OR) for having plasma methylmalonic acid (MMA) concentration >210 nmol/L in logistic regression model.

	Univariable		Multivariable	
	OR (95% Confidence Interval)	*p* Value	Adjusted OR (95% Confidence Interval)	Adjusted *p* Value
Age (years)	0.97 (0.91 to 1.02)	0.25		
Gestational age (weeks)	1.10 (1.03 to 1.17)	0.002	1.11 (1.04 to 1.19)	0.001
Ethnicity				
European	reference	0.001	reference	0.003
Other	0.70 (0.28 to 1.61)		0.59 (0.23 to 1.38)	
Chinese Asian	1.11 (0.47 to 2.45)		1.07 (0.45 to 2.41)	
South Asian	6.10 (2.37 to 16.2)		5.48 (1.95 to 15.8)	
Pre-pregnancy body mass index (kg/m^2^)	1.01 (0.94 to 1.07)	0.84		
Smoking	0.66 (0.10 to 2.48)	0.57		
Education				
Less than high school	reference	0.42		
High school degree	1.20 (0.37 to 4.69)			
University or trade school	0.75 (0.26 to 2.77)			
B12 supplement	0.54 (0.21 to 1.59)	0.25		
Fish intake	0.74 (0.39 to 1.46)	0.38		
Meat intake	0.43 (0.16 to 1.29)	0.12		
Egg intake	0.21 (0.07 to 0.63)	0.006	0.36 (0.10 to 1.28)	0.11

*p* values are from likelihood ratio tests.
